# B5, a thioredoxin reductase inhibitor, induces apoptosis in human cervical cancer cells by suppressing the thioredoxin system, disrupting mitochondrion-dependent pathways and triggering autophagy

**DOI:** 10.18632/oncotarget.5132

**Published:** 2015-09-04

**Authors:** Fang-Yuan Shao, Zhi-Yun Du, Dong-Lei Ma, Wen-Bo Chen, Wu-Yu Fu, Bi-Bo Ruan, Wen Rui, Jia-Xuan Zhang, Sheng Wang, Nai Sum Wong, Hao Xiao, Man-Mei Li, Xiao Liu, Qiu-Ying Liu, Xiao-dong Zhou, Hai-Zhao Yan, Yi-Fei Wang, Chang-Yan Chen, Zhong Liu, Hong-Yuan Chen

**Affiliations:** ^1^ Department of Pathogen Biology and Immunology, School of Basic Course, Guangdong Pharmaceutical University, Guangzhou, P.R. China; ^2^ Guangzhou Jinan Biomedicine Research and Development Center, Guangdong Provincial Key Laboratory of Bioengineering Medicine, National Engineering Research Center of Genetic Medicine, Jinan University, Guangzhou, China; ^3^ Institute of Natural Medicine & Green Chemistry, School of Chemical Engineering and Light Industry, Guangdong University of Technology, Guangzhou, China; ^4^ Department of Biochemistry, Li Ka Shing Faculty of Medicine, The University of Hong Kong, Hong Kong, China; ^5^ University of the Chinese Academy of Sciences, Beijing, China; ^6^ Department of Gastroenterology, The First Affiliated Hospital of Nanchang University, Nanchang, China; ^7^ Center for Drug Discovery, Northeastern University, Boston, MA, USA

**Keywords:** curcumin analog, thioredoxin reductase, cervical cancer, apoptosis

## Abstract

The synthetic curcumin analog B5 is a potent inhibitor of thioredoxin reductase (TrxR) that has potential anticancer effects. The molecular mechanism underlying B5 as an anticancer agent is not yet fully understood. In this study, we report that B5 induces apoptosis in two human cervical cancer cell lines, CaSki and SiHa, as evidenced by the downregulation of XIAP, activation of caspases and cleavage of PARP. The involvement of the mitochondrial pathway in B5-induced apoptosis was suggested by the dissipation of mitochondrial membrane potential and increased expression of pro-apoptotic Bcl-2 family proteins. In B5-treated cells, TrxR activity was markedly inhibited with concomitant accumulation of oxidized thioredoxin, increased formation of reactive oxygen species (ROS), and activation of ASK1 and its downstream regulatory target p38/JNK. B5-induced apoptosis was significantly inhibited in the presence of *N*-acetyl-l-cysteine. Microscopic examination of B5-treated cells revealed increased presence of cytoplasmic vacuoles. The ability of B5 to activate autophagy in cells was subsequently confirmed by cell staining with acridine orange, accumulation of LC3-II, and measurement of autophagic flux. Unlike B5-induced apoptosis, autophagy induced by B5 is not ROS-mediated but a role for the AKT and AMPK signaling pathways is implied. In SiHa cells but not CaSki cells, B5-induced apoptosis was promoted by autophagy. These data suggest that the anticarcinogenic effects of B5 is mediated by complex interplay between cellular mechanisms governing redox homeostasis, apoptosis and autophagy.

## INTRODUCTION

The thioredoxin (Trx) system, composed of thioredoxin reductase (TrxR), Trx, and NADPH, is a major cellular redox control mechanism that is often deregulated in malignancy [1]. The Trx system has special significance in tumor biology as tumor cells are often subject to oxidative stress arising from the tumor environment [2] and increased consumption of reducing equivalents to support DNA synthesis [3, 4]. The Trx system may be involved in carcinogenesis in several ways. Firstly, it can promote cancer cell proliferation through its modulatory effect on redox-regulated transcription factors and/or protein kinase signaling cascades [5]. Secondly, the Trx system is needed for the activity of peroxiredoxins [6], which are ubiquitously expressed antioxidant enzymes that scavenge reactive oxygen species (ROS) [7, 8] and the direct inhibition of apoptosis signal-regulating kinase 1 (ASK1) [9], a mitogen-activated protein kinase (MAPK) kinase kinase (MAPKKK) that activates the p38 and JNK MAPK pathways. As a result, tumor cells usually have higher resistance against oxidative stress-induced apoptosis [10]. Thirdly, the Trx system has also been implicated in tumor angiogenesis [11] through the induction of hypoxia-inducible factor 1 (HIF-1) and vascular endothelial growth factor (VEGF) [12], and the Trx-dependentheme oxygenase-1 (HO-1) pathway [13]. Fourthly, high levels of Trx expression are associated with tumor invasion and metastasis. Thus, Trx promotes matrix metalloproteinase activity and stimulates cancer cell invasion [14]. Trx over expression is linked to tumor metastasis [15] and is implicated in malignant potential of tumor cells [1]. Indeed, Trx levels are upregulated in the plasma of cancerpatients [16] and a high Trx level is often correlated with cancer resistance to chemotherapeutic agents [17–20]. Thus, the Trx system may represent an important therapeutic target in cancer treatment.

**Figure 1:**
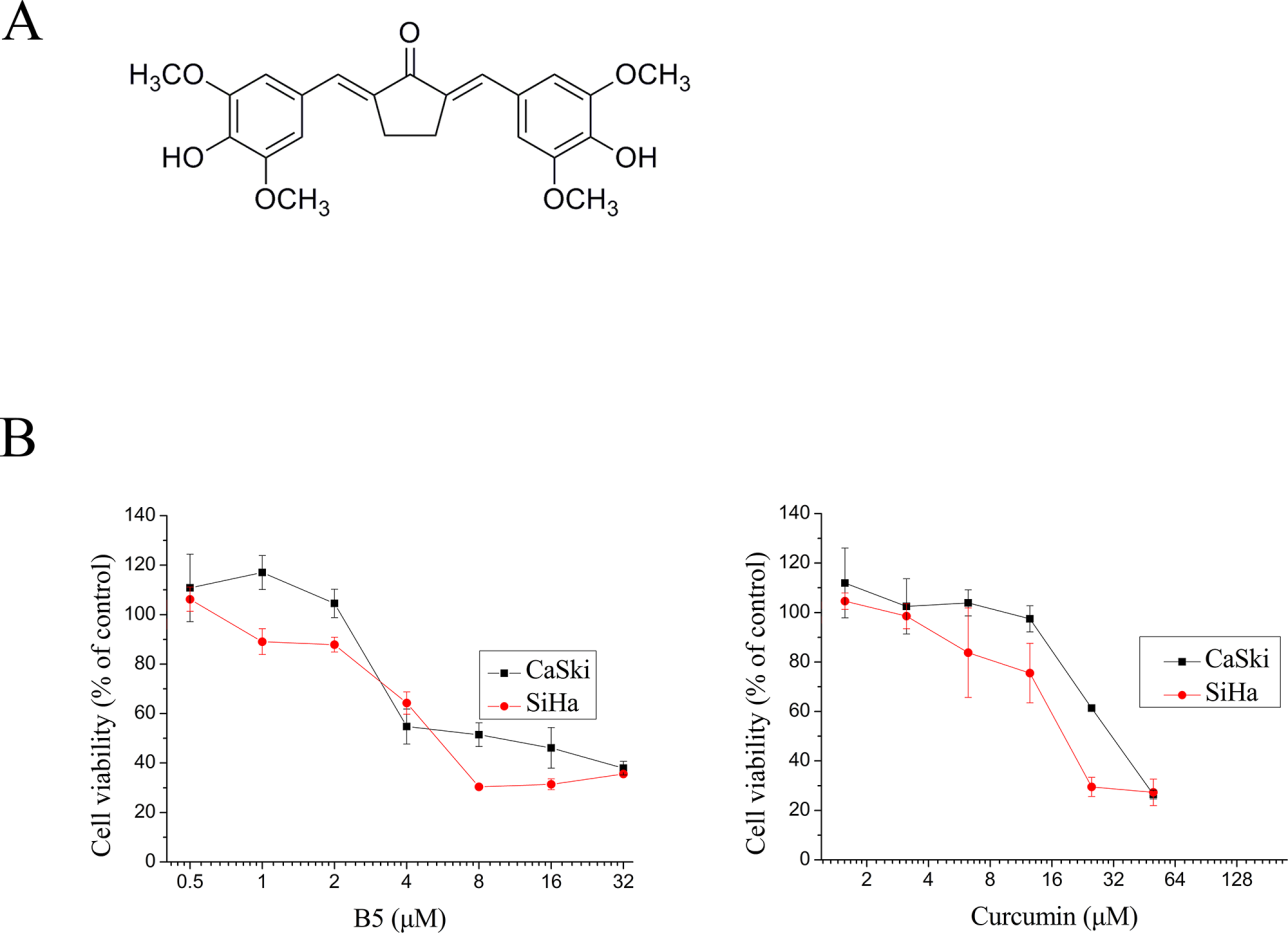
Effects of B5 and curcumin on cell growth **A.** Chemical structure of B5. **B.** Effects of B5 and curcumin on cell growth. CaSki and SiHa cells were treated with the indicated concentrations of B5 or curcumin for 48 h; cell viability was quantified by the MTT assay. Data are the mean ± SD of at least three independent experiments.

TrxR is a ubiquitously expressed selenocysteine (Sec)-containing oxidoreductase that catalyzes the NADPH-dependent reduction of Trx [21]. In our previous study, B5 as one of the synthetic curcumin analogs with structure shown in Figure 1A was found to potently inhibit TrxR by covalent modification of Cys497 and Sec498 [22]. However, the potential anticancer effects of B5 have not been examined. The aim of the present study was to investigate the mechanisms of action of this TrxR inhibitor in two human cervical cancer cell lines CaSki and SiHa.

## RESULTS

### Effects of B5 on the growth of CaSki and SiHa cells

The effect of B5 and on cellular viability in comparison to curcumin was examined in the Caski and SiHa human cervical cancer cells. In MTT assay, the IC_50_ values of B5 and curcumin were respectively 6.1 and 28.3 μM in CaSki cells. In SiHa cells, the IC_50_ values were 5.3 and 18.5 μM, respectively (Fig. 1B). These results show that B5 is relatively more cytotoxic towards cancer cells than natural curcumin.

### B5 induces cell cycle arrest and apoptosis in CaSki and SiHa cells

**Figure 2:**
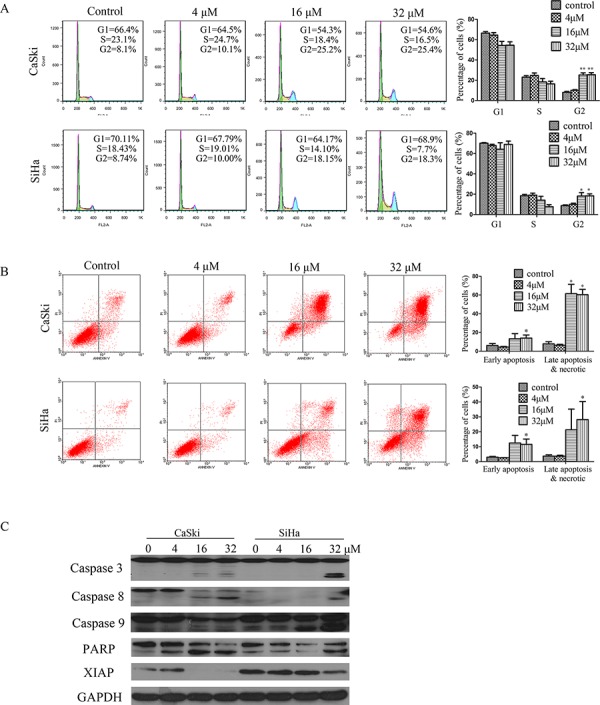
B5 induced apoptosis and cell cycle arrest in CaSki and SiHa cells **A.** Effects of B5 on cell cycle distribution. Cells were treated with 0, 4, 16, and 32 μM B5 for 48 h, fixed in 70% ethanol, stained with PI, and cell cycle distribution was assessed by flow cytometry. The percentage of cells in each cell cycle phase is indicated as the mean ± SD of three independent experiments. **B.** Induction of apoptosis by B5 in CaSki and SiHa cells. Cells were treated as above and analyzed by flow cytometry after Annexin-V-FITC/PI staining; data are presented as the mean ± SD of three independent experiments. *P < 0.5 and **P < 0.01. **C.** Western blotting analysis of the expression of the following: pro- and cleaved-caspases 3, 8, and 9; PARP (86kDa); and XIAP. Data are representatives of three independent experiments.

To study the mechanism underlying the inhibitory effects of B5 on cancer cell growth, cell-cycle analysis was performed for B5-treated CaSki and SiHa cells by flow cytometry. The treatment of cells with B5 (16 μM) resulted in an increase in the percentage of cells in the G2/M phases by18% and 10% respectively in the CaSki and SiHa cells when compared to the controls. A similar increase in the percentage of G2/M cells was again observed after treating the cells with B5 at 32 μM (Fig. 2A).

Whether the treatment of cells with B5 would also result in apoptosis was next examined. Cells were stained with a mixture of Annexin-V-FITC/PI followed by flow cytometry analysis. B5 at 16 and 32 μM caused readily detectable cell death to occur in both cell lines as evident by the presence of cells stained positively with Annexin V-FITC only (early apoptotic) or Annexin-V-FITC/PI (late apopotic). In the CaSki cells, B5 at 16 and 32 μM caused a small but distinct increase in the percentage of early apoptotic cells. By contrast, a rather large increase in the percentage of late apoptotic cells amounting to 58.7% and 60.5% respectively at 16 and 32 μM of B5 was observed. Similarly, more late than early apoptotic cells were formed in B5-treated SiHa cells. At B5 concentrations of 16 and 32 μM, the percentage late apoptotic cells was approximately double that of the untreated control.

We next analyzed the effect of B5 on the activation of caspases and expression of XIAP. Western blotting analysis showed that B5 induced the activation of caspase 3, caspase 8, and caspase 9. Consistent with the activation of caspases, the caspase 3 substrate PARP was found to undergo specific proteolytic cleavage as suggested by the presence of the 116 kDa to 89 kDa fragment in cells treated with B5 at 4, 16 and 32 μM in CaSki cells. In the case for SiHa cells, an increase in the abundance of the 89 kDa PARP fragment could readily be seen in cells treated with B5 at 32 μM (Fig. 2C). In addition, B5 treatment downregulated the expression of XIAP (Fig. 2C), which is considered the most potent human IAP protein currently identified because it inhibits the activity of both caspase 3 and caspase 9 [23, 24]. These results suggest that caspases activation may underlie the apoptotic activity of B5 in cervical cancer cells.

### B5 induces mitochondrial dysfunction and regulates the expression of Bcl-2 family proteins

**Figure 3:**
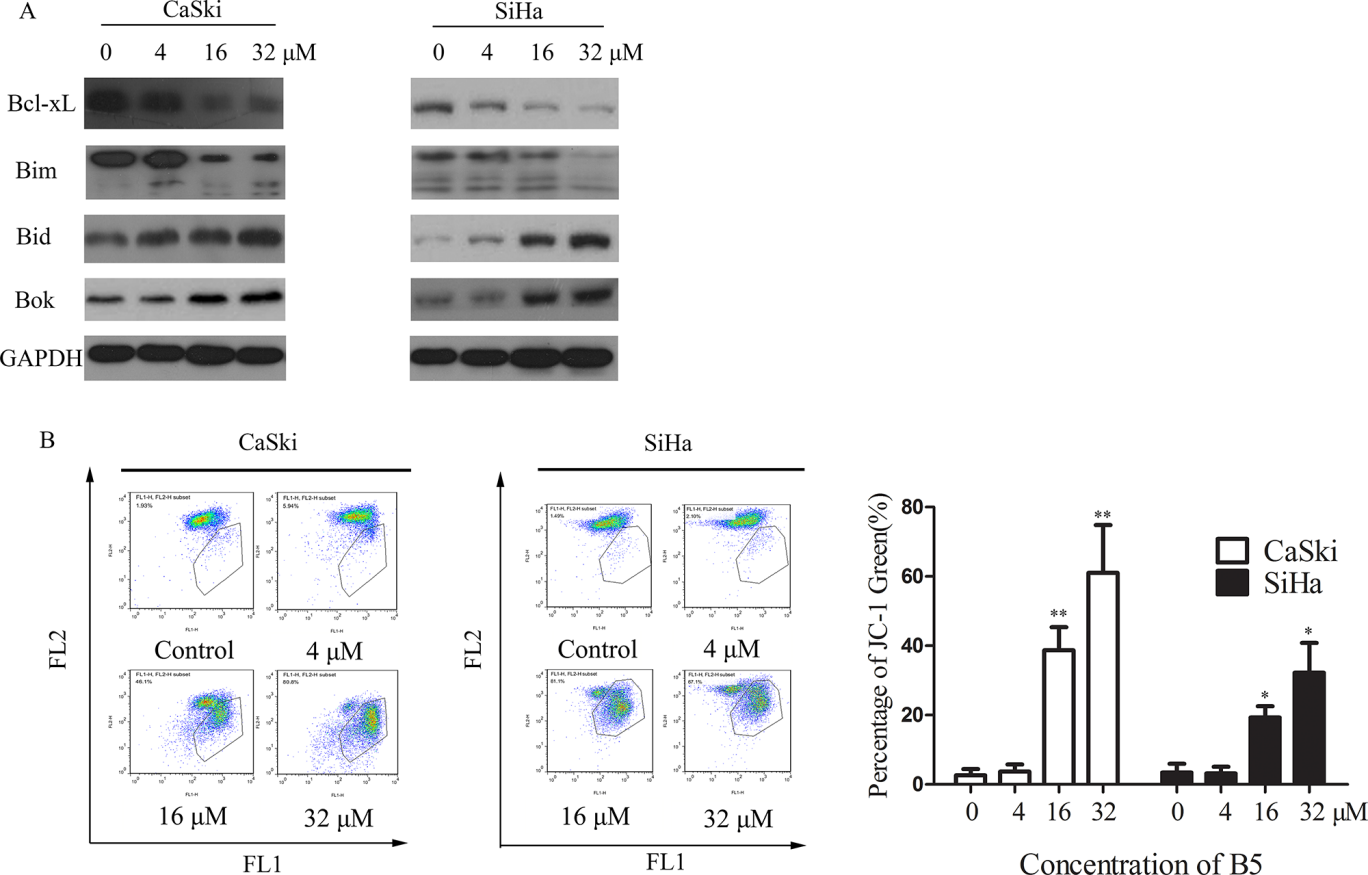
Effects of B5 on the Bcl-2 family proteins and mitochondrial membrane potential (MMP) **A.** Expression of the Bcl-2 proteins was analyzed by western blotting, with GAPDH used as the internal control. **B.** Flow cytometry analysis of MMP by JC-1 staining. Cells were treated with 0, 4, 16, and 32 μM B5 for 48 h and stained with JC-1 for 20 min. Cells with MMP loss were gated. Data are presented as the mean ± SD of three independent experiments. *P < 0.5 and **P < 0.01.

A distinctive feature of the early phase apoptosis is a change in mitochondrial membrane potential (Δψ_m_) [25] that is an important parameter of mitochondrial function. The Δψ_m_ is an early event preceding caspase activation, and is regarded as a hallmark of apoptosis [26]. Therefore, we measured Δψ_m_ in B5-treated CaSki and SiHa cells using the membrane-permeable JC-1 dye. In apoptotic or unhealthy cells with low Δψ_m_, JC-1 remains in the monomeric form, which has green fluorescence [27]. As shown in Fig. 3B, a marked increase in JC-1-related green fluorescence can be seen in both the CaSki and SiHa cells treated with 16 or 32 μM of B5. These results demonstrate that B5 induced MMP disruption in both cell lines.

Mitochondrion-mediated intrinsic apoptotic pathway occurs in response to various stimuli, including oxidative stress, and is regulated by the proteins of the Bcl-2 family [28, 29]. In this study, we found that B5 induced downregulation of antiapoptotic Bcl-xL, upregulation of proapoptotic Bid/Bok, and activation of Bim cleavage in CaSki cells (Fig. 3A). However, the expression of Bax and Bcl-2 was not affected by B5 (data not shown). These results indicate that B5 induces apoptosis of cancer cells through induction of mitochondrial dysfunction caused by deregulation of Bcl-2 family proteins.

### Effect of B5 on ROS generation

**Figure 4:**
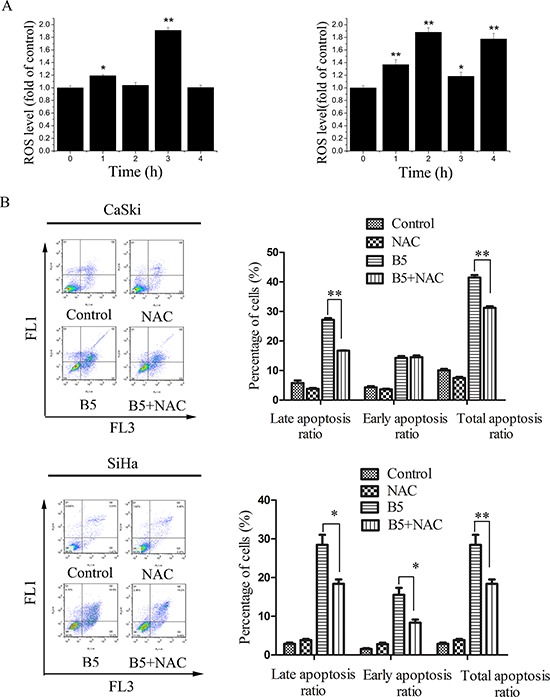
The role of ROS production in B5-induced cell apoptosis **A.** B5-induced ROS production in CaSki (left) and SiHa (right) cells. Cells were treated with 16 μM B5 for 1–4 h, stained with DCFH-DA for 20 min, and analyzed for fluorescence at 525 nm. **B.** B5-induced CaSki and SiHa cell apoptosis was inhibited by NAC pre-treatment. Cells were pre-treated with 10 mM NAC for 30 min and treated with 16 μM B5 for 48 h; then, they were stained with Annexin-V-FITC/PI and analyzed by flow cytometry. All values are presented as the mean ± SD of three independent experiments. *P < 0.5 and **P < 0.01.

ROS have important roles in intracellular signal transduction and redox homeostasis [30]. However, excessive ROS accumulation can exert toxic effects and cause oxidative stress, damaging main cellular components such as DNA, lipids, and proteins [31] and causing apoptosis [32]. To investigate whether B5-induced apoptosis was related to the changes in intracellular redox environment, we examined intracellular ROS production in B5-treated cervical cancer cells using DCFH-DA. As shown in Fig. 4A, B5 caused ROS accumulation in CaSki and SiHa cells, especially after the treatment for 3 and 2 h, respectively. The pre-treatment of NAC, a ROS scavenger, reduced B5-induced apoptosis by 10.5% and 13.4%, respectively (Fig. 4B). Together, these results suggest that ROS accumulation plays an important role in B5-induced cell apoptosis.

### Effect of B5 on the Trx system

**Figure 5:**
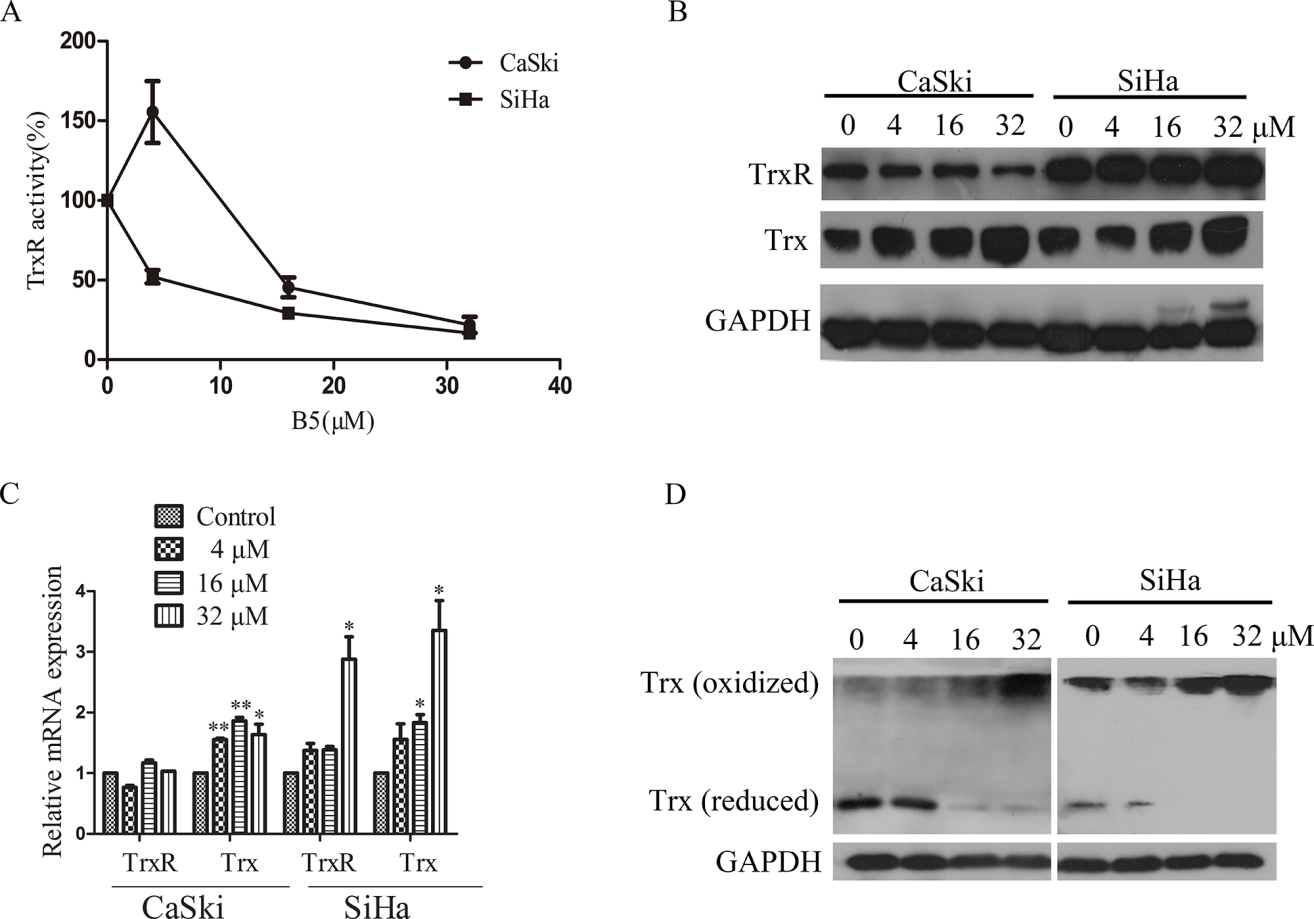
Effects of B5 on the Trx system Cells were treated with 0, 4, 16 and 32 μM B5 for 48 h. **A.** TrxR activity in cell lysates was determined by SC-TrxR assay. Data were presented as the percentage of control. **B.** Trx and TrxR protein levels were analyzed by SDS-PAGE and western blotting. **C.** The mRNA levels of Trx and TrxR were measured by RT-PCR; GAPDH was used as an internal control. **D.** Trx redox status was determined by non-reduced SDS-PAGE and western blotting analysis; GAPDH was used as the internal control. All data are representatives of three independent experiments.

We next examined B5 effects on the Trx system. SC-TrxR assay showed that B5 inhibited TrxR activity dose-dependently in both cell lines (Fig. 5A). Interestingly, the expression level of Trx was increased in both cell lines, whereas that of TrxR was increased in SiHa cells but decreased in CaSki cells (Fig. 5B). This result was further verified by the RT-PCR assay: B5 upregulated Trx mRNA in both cell lines, while TrxR mRNA was upregulated only in SiHa cells (Fig. 5C). Nevertheless, the oxidized Trx form was increased while the reduced form was decreased dose-dependently in both cell lines after the treatment with B5 (Fig. 5D). Together, these results indicate that B5 deregulates the Trx system in cancer cells.

### Effect of B5 treatment on the MAPK signaling in cervical cancer cells

**Figure 6:**
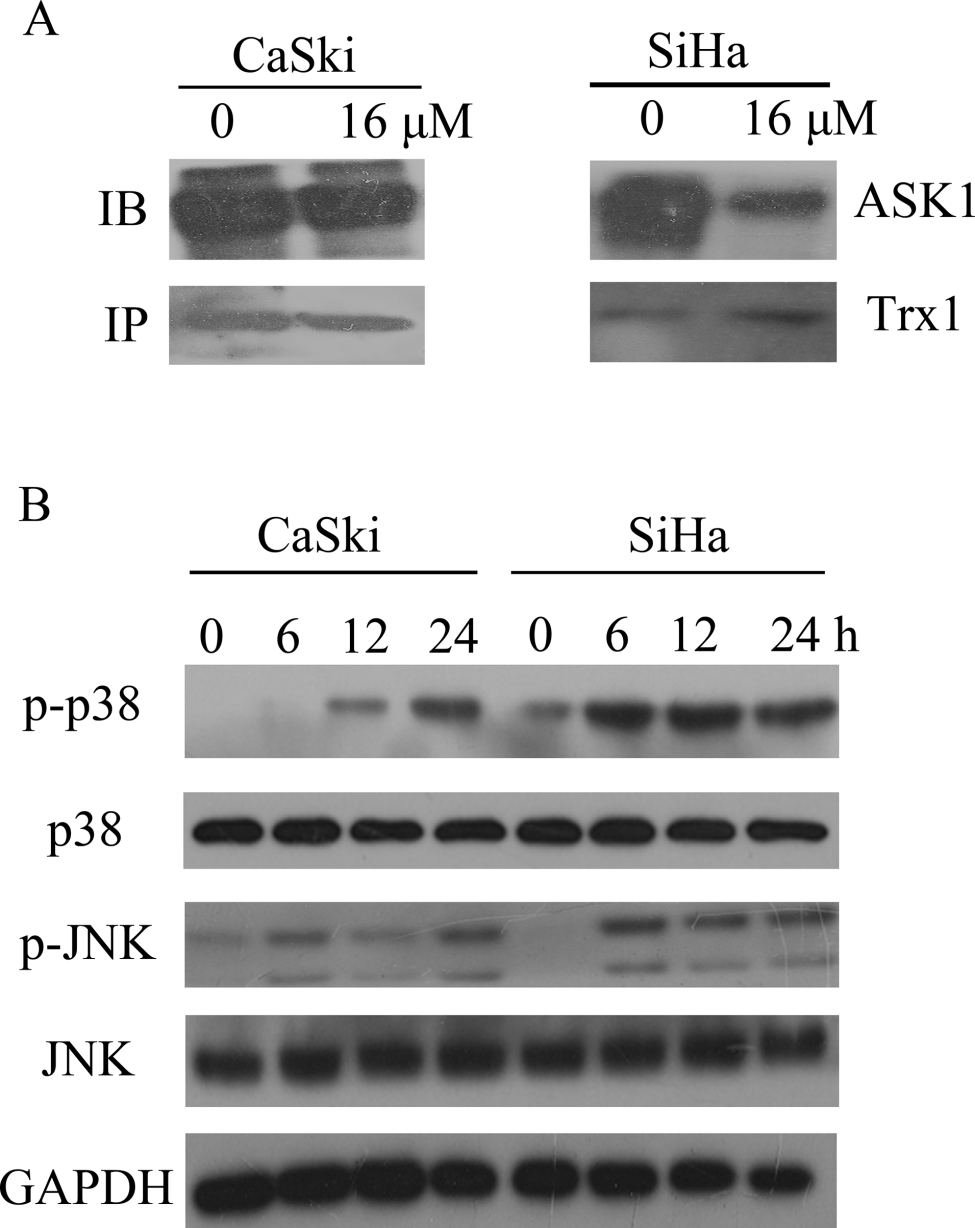
Effects of B5 on ASK1 and p38/JNK MAPKs **A.** CaSki and SiHa cells were treated with 16 μM B5 for 8h; ASK1-Trx complex was precipitated using Protein A beads and analyzed by western blotting with ASK1 and Trx antibodies. **B.** Activation of p38/JNK MAPKs. Cells were treated with 16 μM B5 for 0, 6, 12 and 24 h and subjected to western blotting analysis; GAPDH was used as the internal control.

The inhibition of TrxR activity should result in the shift of Trx redox state to the oxidized form (Fig. 5C) and dissociation of ASK1 from the complex with Trx [10]. In our study, co-immunoprecipitation assay showed that B5 induced the release of ASK1 from the complex with Trx (Fig. 6A). The increased presence of free ASK1 was also indicated by the activation of downstream MAPK signaling pathways mediated by ASK1: the phosphorylation level of p38 and JNK MAPKs notably increased in a time-dependent manner (Fig. 6B), suggesting activation of these kinases in CaSki and SiHa cells after B5 treatment.

### B5 induces autophagy in cervical cancer cells

**Figure 7:**
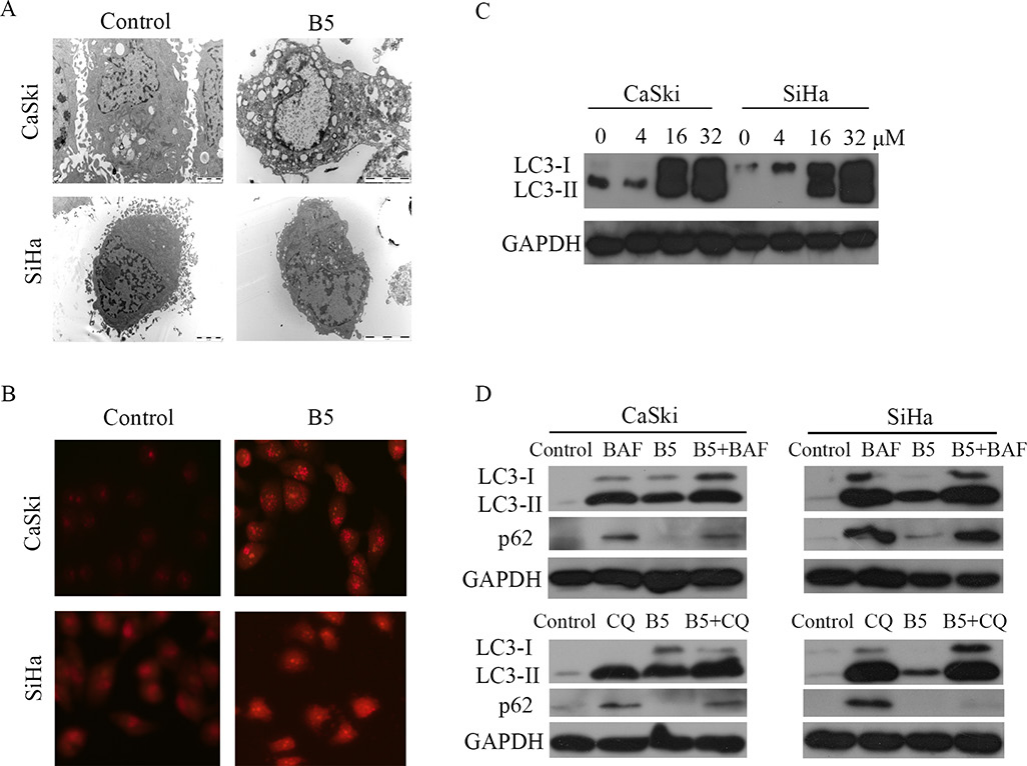
B5-induced autophagy in CaSki and SiHa cells **A.** Ultrastructure of CaSki and SiHa cells with the treatment of 16 μM B5 for 48 h; transmission electron microscopy shows multiple autophagic vacuoles. **B.** Cells were treated with B5 for 48 h and then stained with acridine orange and examined under a fluorescence microscope at magnification of 400. **C.** Western blotting analysis of LC3 expression in CaSki and SiHa cells with the treatment of 0, 4, 16, and 32 μM B5 for 48 h. **D.** Autophagic flux analysis by measuring the expression levels of SQSTM1/p62 or LC3-II. CaSki and SiHa cells were treated with BAF or CQ prior to 16 μM B5 treatment for 48 h. Expression levels of SQSTM1/p62 and LC3-II were determined by western blotting analysis.

It is unclear whether autophagy in dying cells is the cause of cell death or is actually an attempt to prevent it. Autophagy-related cell death has been defined as a form of programmed cell death [33]. The treatment with 16 μM B5 for 48 h induced autophagy in CaSki and SiHa cells, as demonstrated by the presence of autophagic vacuoles by electron microscopy (Fig. 7A). To confirm B5-induced autophagy, acridine orange, a lysosome marker dye, was used to stain CaSki and SiHa cells [34]. As shown in Fig. 7B, B5 induced the accumulation of acridine orange in cytoplasm of CaSki and SiHa cells, while less accumulation of acridine orange was observed in control cells. Furthermore, B5 also induced accumulation of LC3-II (Fig. 7D), a lipidated form of LC3 that is regarded as an autophagosomal marker in mammals. To obtain further confirmation of autophagy, autophagic flux was measured in terms of alteration of LC3-II and SQSTM1/p62 levels in the presence and absence of specific lysosomal inhibitors BAF and CQ that act to prevent autophagosomal lysosome degradation [35]. As shown in Fig. 7D, BAF or CQ treatment resulted in accumulation of LC3-II and SQSTM1/p62 both in CaSki and SiHa cells. The accumulation of LC3-II was increased and that of SQSTM1/p62 was decreased with the combination of inhibitors with B5. These results suggest that B5 induces autophagy in cervical cancer cells.

**Figure 8:**
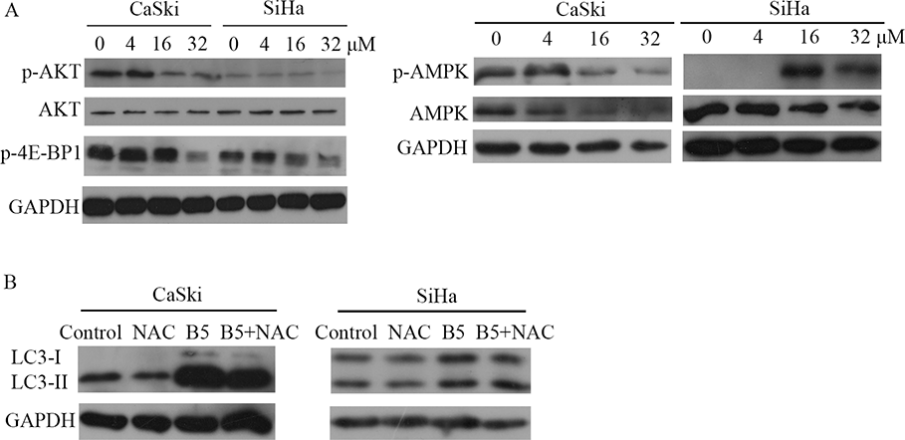
A. Expression levels of p-AKT, AKT, p-4E-BP1, AMPK and p-AMPK in CaSki and SiHa cells Cells were treated with 0, 4, 16, and 32 μM B5 for 48 h and subjected to western blotting analysis. GAPDH was used as the internal control. **B.** Effect of ROS on B5-inducd autophagy. CaSki and SiHa cells were treated with ROS inhibitor NAC prior to 16 μM B5 treatment for 48 h. Expression levels of LC3-II were determined by western blotting analysis. All data are representatives of three independent experiments.

The AKT-mediated signaling cascade is one of the main signaling pathways regulating autophagy [36]. Figure 8A showed that the phosphorylation level of AKT was declined after B5 treatment, suggesting the involvement of the AKT signaling in B5-induced autophagy. The energy sensor AMPK has recently been connected to autophagy [37]. Interestingly, the phosphorylation level of AMPK was promoted in SiHa cells, but it was suppressed in CaSki cells with B5 treatment (Fig. 8A). A complex role of AMPK in B5-mediated autophagy in cervical cancer cell lines is implied. TORC1 is a downstream regulatory target of AKT and AMPK and it usually acts as an autophagy-suppressive regulator. TORC1 activity can be followed by the phosphorylation of its substrate 4E-BP1 [35, 38]. As shown in Fig. 8A, the phosphorylation level of 4E-BP1 was decreased in both cell lines upon treatment with B5.

A growing amount of evidences in recent years demonstrate that ROS represent important mediators of autophagy [39]. To investigate the role of ROS in B5-inducd autophagy, CaSki and SiHa cells were pretreated with NAC prior to B5 treatment. As shown in Fig. 8B, LC3-II accumulation was not affected by NAC, suggesting that B5-mediated autophagy induction was independent on B5-caused ROS production in cervical cancer cells.

### Effect of B5-induced autophagy on B5-triggered apoptosis in cervical cancer cells

**Figure 9:**
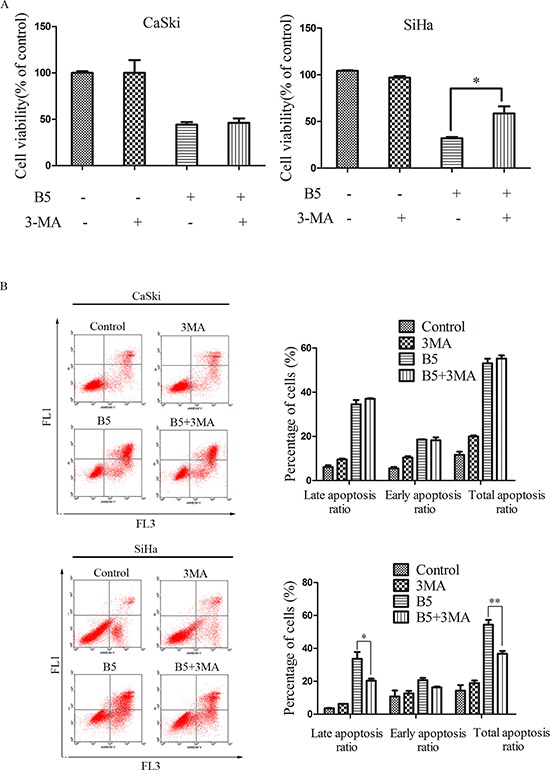

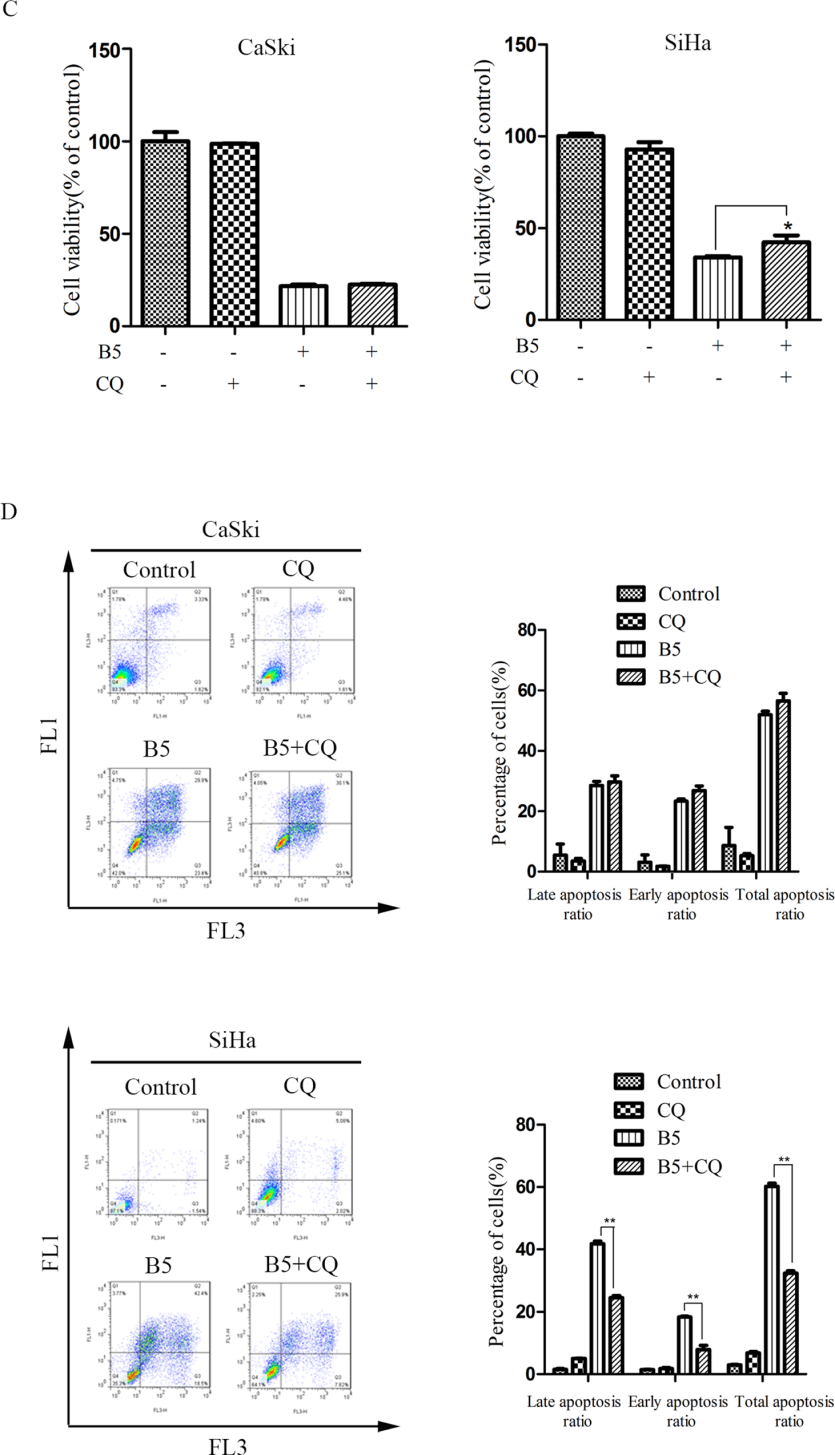
Effects of the autophagy inhibitors, 3-MA and CQ, on B5-induced cell growth inhibition and apoptosis The cells were pretreated with 5 mM 3-MA **A.** and **B. C.** and **D.** For 1 h and then treated with 16 μM B5 for 48 h. Cell viability was determined by the MTT assay (A and C). Apoptotic cells were quantified by Annexin V-FITC/PI staining analysis (B and D). Data are presented as the mean ± SD of three independent experiments. *P < 0.5 and **P < 0.01.

To explore whether B5-induced autophagy had a protective or detrimental effect, CaSki and SiHa cells were pre-cultured with two different autophagy inhibitors, 3-MA and CQ, respectively, before the treatment of B5. The MTT assay indicated that 3-MA, a commonly used inhibitor of starvation or rapamycin-induced autophagy [40], prevented the inhibitory effect of B5 on the growth of SiHa cell lines (Fig. 9A right), suggesting that B5-induced autophagy may have an apoptosis-promoting effect. To verify this hypothesis, flow cytometry was used to analyze cellular apoptosis induced by the combination of B5 and 3-MA. The results indicate that B5-induced autophagy indeed had an apoptosis-promoting effect but only in SiHa cells (Fig. 9B bottom).

It has been reported that 3-MA might also inhibit PI3K and can thus promote autophagy in some systems as well as affect cell survival [41]. Another autophagy inhibitor CQ was therefore used to further examine the involvement of autophagy in B5-triggered apoptosis by MTT assay and flow cytometry analysis. As shown in Fig. 9C and 9D, CQ prevented the growth inhibitory effect of B5 and attenuated B5-triggered apoptosis in SiHa cells. However, no obvious changes of growth and apoptosis were observed in CaSki cells when B5 was combined with CQ. These results suggest that B5-induced autophagy has different roles in B5-induced apoptosis in CaSki and SiHa cells.

## DISCUSSION

**Figure 10:**
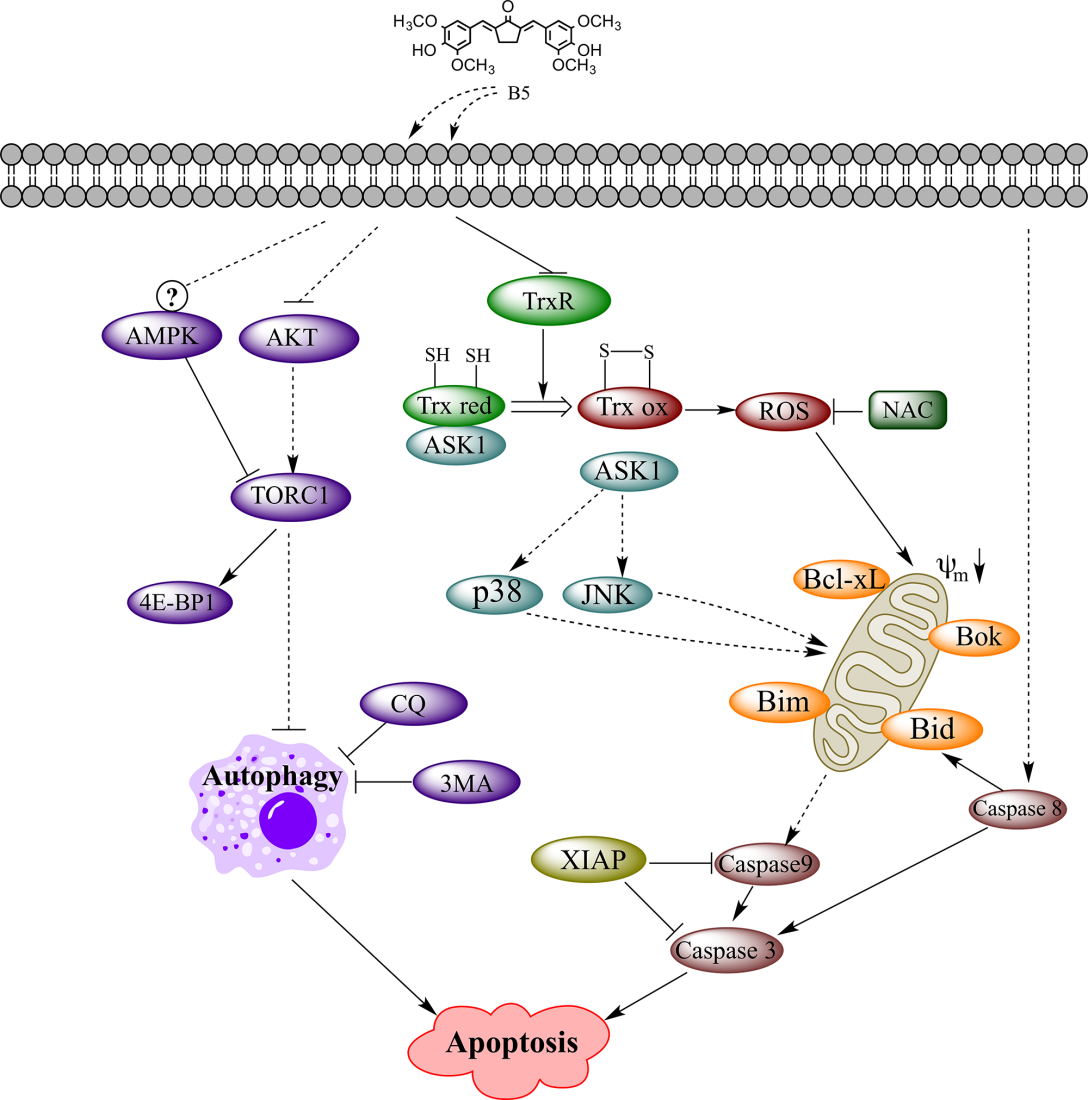
TrxR inhibitor B5-mediated intracellular signaling that led to apoptosis in human cervical cancer cells Key: black arrows, activation; black blocks, inhibition; dashed arrows or blocks, indirect interaction; circled question mark, complex regulation.

The mammalian TrxR is a Sec-containing oxidoreductase that has been established as an anticancer drug target [4, 42, 43] because of its role as a key player in cell death program [44]. The highly reactive Sec active site with low pKa value and easily accessible location makes this enzyme a suitable target for drug development. In the past decades, many therapeutically used synthetic and natural compounds with TrxR inhibitory activity have been found to exhibit anticancer potential [45]. Among them, gold compounds [46], platinum drugs [47], flavonoids [48], quinines [49], mercury [50], arsenic trioxide [51], curcumin [52] and its analogs [22] have been tested as novel cancer treatments or adjuncts to existing therapy; some of them have shown potent therapeutic effects [53]. In the present study, we demonstrated that B5, a newly synthesized TrxR inhibitor and a curcumin analog, exhibited significant antitumor activity based on the modulation of the intracellular redox status and autophagy (Fig. 10).

Mitochondria plays a very important role as the intrinsic apoptosis pathway [54]. The key event of mitochondrion-induced apoptosis is the permeabilization of the OMM, which occurs in response to various stimuli and is mainly regulated by the members of the Bcl-2 family [28, 29]. Indeed, we found that B5 decreased Δψ_m_ in CaSki and SiHa cells (Fig. 3B) and downregulated the expression of antiapoptotic Bcl-xL while upregulating that of proapoptotic Bid/Bok (Fig. 3A), indicating that the loss of Δψ_m_ plays an important role in B5-induced apoptosis in cervical cancer cells.

MMP loss is an early event preceding caspase activation, which causes the leakage of small molecules, such as cytochrome *c* and second mitochondria-derived activator of caspases (Smac), from the mitochondria into the cytoplasm [55], followed by the (a) recruitment of apoptotic protease activating factor-1 (Apaf-1) and procaspase9 to form the apoptosome [56], (b) activation of caspase 9 and caspase 3, and (c) execution of apoptosis [57]. The inhibitor of apoptosis (IAP) family negatively regulates this process, and the most potent human IAP protein XIAP inhibits the activity of both caspase 9 and caspase 3 [58]. Our data demonstrate that B5 induced the activation of caspases 3, 8, and 9 in CaSki and SiHa cells (Fig. 2C) and downregulated the expression of XIAP (Fig. 2C), indicating that B5 induced apoptosis in cancer cells through the intrinsic as well as extrinsic apoptotic pathways as evidenced by the activation of caspase 8 [43].

The inhibition of TrxR activity results in the accumulation of ROS. In healthy cells, ROS are natural byproducts of metabolism and have important roles in cell signaling and homeostasis. However, excessive ROS generation can damage cell structures and cause oxidative stress and cell death [31]. In our experiments, B5 induced time-dependent ROS accumulation (Fig. 4A) suppression in TrxR activity and increase in the oxidized Trx form (Fig. 5A and 5D) in cancer cells; furthermore, the ROS scavenger NAC reduced B5-elicited apoptosis (Fig. 4B), suggesting that B5-dependent disruption of the Trx system and excessive ROS accumulation underlie the proapoptotic activity of B5. It should be noted that a decrease of ROS level was observed in CaSki and SiHa cells after the treatment of B5 for 4 and 3 h, respectively. It may be that some cellular antioxidant proteins, such as peroxiredoxins, glutathione peroxidase, and catalase, participated in preventing the buildup of intracellular ROS [59].

MAPK signaling pathways are closely involved in the response to oxidative stress [60]. JNK and p38 MAPKs play important roles in regulating H_2_O_2_-induced cell death and are critical mediators of oxidative stress-induced apoptosis [61, 62]. The binding of reduced Trx to the upstream kinase ASK1 can block JNK and p38 activity; however, when Trx is oxidized, the complex dissociates and the released ASK1 activates the JNK and p38 pathways, thereby promoting apoptosis. Our results show that B5 treatment causes ASK1 dissociation from the complex with Trx in SiHa cells (Fig. 6A) and activation of JNK and p38 MAPKs (Fig. 6B), suggesting that MAPK pathway may be involved in B5-induced apoptosis. The more detailed role of MAPK pathway in B5-induced apoptosis and the relationship between B5-mediated ROS production and MAPKs signal transduction are worthy of further investigation.

Although autophagy can be upregulated in many tumor cells under stress conditions such as chemotherapy or nutrient deficiency, the exact role of autophagy in cancer cell death is still not clear [63, 64]. After short-term stressful effects, autophagy activation generally facilitates the restoration of cell homeostasis and survival. However, under permanent adverse conditions, autophagy is induced to promote elimination of damaged cells by apoptosis [33]. Here, we investigated the association between apoptosis and autophagy in cervical cancer cells treated with the combination of B5 and the autophagy inhibitors. The reduction in autophagy relieved B5 inhibitory effect on cell growth (Fig. 9A and 9C), which is verified by Annexin V-FITC/PI staining analysis in SiHa cells (Fig. 9B and 9D), suggesting a crosstalk between B5-induced apoptosis and autophagy in SiHa cells. However, the treatment of autophagy inhibitors, both 3-MA and CQ, had no effect on B5-induced apoptosis in CaSki cells (Fig. 9), suggesting different signaling pathways may be involved in B5-mediated autophagy in CaSki and SiHa cell lines.

Indeed, in this study, we found that AKT signaling, one of the main signaling pathways regulating autophagy, was inconsistently inhibited in CaSki and SiHa cells (Fig. 8A). Although the phosphorylation level of AKT was inhibited markedly at high concentration (32 μM) of B5 in both cell lines, it was hardly reduced by 16 μM B5 in SiHa cells in spite of autophagy induction under the treatment of this concentration, indicating the involvement of some other pathway(s) in the regulation of B5-induced autophagy in SiHa cells. Herein AMPK was found to one of the possible regulators. There is mouting evidence that AMPK activates autophagy through inactivation of TORC1 and direct phosphorylation of the protein kinase ULK1 [37]. Our results in Figure 8A that the phosphorylation level of AMPK was elevated dramatically by B5 in SiHa cells suggests that B5 may induce autophagy in SiHa cells through coordinated regulation of AKT and AMPK followed by the suppression of TORC1, whose activity was monitored by the decreased phosphorylation level of 4E-BP1 (Fig. 8A), a substrate of TORC1. It is interesting to note that some unexpected result was observed in CaSki cells, namely the level of phosphorylated AMPK was decreased, which was inconsistent with that of SiHa cells. It may be that more complex network of regulation are involved in B5-mediated autophagy in CaSki cells since the activation of AMPK crucially depends on phosphorylation by multiple upstream kinases, like LKB1, CaMKK, TAK1, etc [65]. The detailed roles of the regulation network in B5-mediatd autophagy and crosstalks and feedbacks between B5-induced apoptosis and autophagy remain to be further explored.

In summary, we demonstrated that B5 activated apoptosis in cervical cancer cells via suppression of Trx system and functional disruption of mitochondria. The accumulation of the oxidized Trx would cause ROS accumulation and affect a number of Trx-dependent pathways, including those of ASK1, JNK and p38 MAPKs, which may contribute to B5-induced apoptosis. B5 also promoted apoptosis by induction of autophagy, which might involve the inhibition of the Akt signaling and was independent of ROS (Fig. 10). Our results show that B5 is a more efficient anticancer agent than curcumin *in vitro* and warrants further *in vivo* investigation. Our data provide important information for the explanation of the anticancer activity of curcumin analogs and for the development of new curcumin analogs targeting TrxR with high activity against cancer cells.

## MATERIALS AND METHODS

### Cell culture and reagents

The human cervical cancer cell lines CaSki and SiHa were purchased from the Cell Bank of the Chinese Academy of Sciences (Shanghai, China) and stored in liquid nitrogen. Cells were cultured in DMEM (Gibco, USA) containing 10% fetal bovine serum (FBS, Gibco, USA), 100 U/mL penicillin, and 100 μg/ml streptomycin (complete medium) at 37°C in a humidified 5% CO_2_ atmosphere.

Curcumin was isolated from *Curcuma longa* roots and purified by silica gel chromatography. B5 was synthesized in our laboratory as described in a previous report [66].

Dimethylsulfoxide (DMSO), 3-methyladenine autophagy inhibitor (3-MA), 5, 5′, 6, 6′-tetrachloro-1, 1′, 3, 3′-tetraethyl-imidacarbocyanine iodide (JC-1), *N*-acetyl-l-cysteine (NAC), 2′, 7′-dichlorofluoresceindiacetate (DCFH-DA), selenocystine (SC), acridine orange, chloroquine (CQ), bafilomycin A_1_ (BAF), and 3-(4, 5-dimetrylthiazol-2-yl)-2, 5-diphenyltetrazolium bromide (MTT) were purchased from Sigma (St. Louis, MO, USA). Propidium iodide (PI), RNase and RIAP buffer kit containing protease inhibitors cocktail (phenylmethanesulfonyl fluorideand and leupeptin) and phosphatase inhibitors cocktail (sodium fluoride and sodium orthovanadate) were purchased from Beyotime (Shanghai, China).

Antibodies against the following were purchased from Cell Signaling Technology (Beverly, MA, USA): ASK1; GAPDH; Bax; caspases 3, 8, and 9; X-linked inhibitor of apoptosis (XIAP); PARP; p38; p-p38 MAPK (Thr180/Tyr182); AKT; p-AKT (Ser473); JNK; p-JNK1/2 (Thr183/Tyr185); p-4E-BP1; LC3; AMPK; p-AMPK and SQSTM1/p62. Trx1, TrxR and horseradish peroxidase-conjugated secondary antibodies were purchased from Santa Cruz Biotechnology (Santa Cruz, CA, USA).

### Growth inhibition assay

Growth inhibition of cancer cells by B5 was measured by the MTT assay. Exponentially grown CaSki and SiHa cells were seeded in 96-well plates at the density of 5 × 10^3^ cells per well in complete medium and allowed to attach for about 12 h. Cells were then treated with a range of B5 concentrations for 48h. The medium was removed, and 50 μl of the same medium containing 5 mg/ml MTT was added to each well for another 4 h. After medium removal, 100 μl DMSO was added to each well for 10 min. Cell viability was assessed by the absorbance at 570 nm and measured using a microplate reader (BIO-RAD, USA); IC_50_ values of B5 for each cell line were determined by comparison with untreated control cells.

### Flow cytometry analysis of cell cycle arrest and apoptosis

CaSki and SiHa cells (2 × 10^5^) were seeded in 60-mm Petri dishes at 1 day before the experiment. For cell cycle analysis, cells were harvested, washed twice with ice-cold PBS, and fixed in 70% ethanol at 4°C overnight after treatment with 4, 16, and 32 μM B5 for 48 h. Then cells were washed once with ice-cold PBS and re-suspended in 1 mL of staining reagent containing 50 mg/mL PI and 100 mg/mL RNase for 30 min in the dark. To assess apoptosis, harvested cells were washed twice with ice-cold PBS, and stained with Annexin-V-FITC/PI (KeyGEN, Nanjing, China) according to the manufacturer's instructions. Cell cycle arrest and apoptosis were analyzed by flow cytometry (BD FACSCalibur, USA). Fluorescence of Annexin-V-FITC and PI was monitored at 630 nm and 525 nm, respectively.

### Evaluation of mitochondrial membrane potential (MMP)

CaSki and SiHa cells (2 × 10^5^) were seeded in 60-mm Petri dishes at 1 day before the experiment. After the treatment with 4, 16, and 32 μM B5 for 48 h, cells were harvested, washed twice with ice-cold PBS, and incubated with JC-1 (10 μg/ml) in the dark for 15 min at 37°C. Cells were washed three times with ice-cold PBS and analyzed by flow cytometry using emission wavelengths of 590 nm and 525 nm.

### Determination of ROS production

CaSki and SiHa cells (2 × 10^5^) were seeded in 60-mm Petri dishes. After the treatment with 16 μM B5 for 1–4 h, cells were incubated with 10 μM DCFH-DA for 15 min in the dark, washed three times with ice-cold PBS, and seeded in 96-well NUNC plates (Thermo, USA) at the density of 5 × 10^3^ per well; DCFH-DA fluorescence was measured at 525 nm using a microplate reader (Tecan F500, Switzerland).

### Transmission electron microscopy analysis

CaSki and SiHa cells (2 × 10^5^) were seeded in 60-mm Petri dishes at 1 day before the experiment. After the treatment with 16 μM B5 for 48 h, cells were harvested, washed twice with ice-cold PBS, resuspended in 70% Karnovsky's fixative, and incubated for 20 min at room temperature. Ultrathin slices were prepared and examined under Hitachi 7000 transmission electron microscope (Tokyo, Japan).

### Acridine orange staining

CaSki and SiHa cells were treated as described in transmission electron microscopy analysis. After harvesting, cells were washed twice with ice-cold PBS and incubated with 1 μg/ml acridine orange for 15 min at 37°C. Subsequently, cells were washed three times with ice-cold PBS and then observed under a fluorescence microscope (Olympus IX71, Japan).

### Immunoblotting and immunoprecipitation

After treatment with different concentrations of B5 for 48 h, cells were harvested and washed twice with ice-cold PBS. For immunoblotting analysis, cell lysates were prepared by incubation in RIPA buffer containing protease inhibitors cocktail (1 mM phenylmethanesulfonyl fluorideand and 1 μg/ml leupeptin) and phosphatase inhibitors cocktail (1 mM sodium fluoride and 1 mM sodium orthovanadate) for 30 min on ice and centrifuged at 12,000 rpm for 15 min. Supernatants were collected, and equal amounts of denatured proteins (heat samples to 95°C for 5 min) were resolved in SDS-PAGE gels. Proteins were transferred to nitrocellulose membranes, blocked with 5% nonfat milk at room temperature for 1 h, and incubated with primary antibodies overnight at 4°C. The membranes were washed three times with Tris-buffered saline-5% Tween 20 (TBST) solution and incubated with a horseradish peroxidase-conjugated secondary antibody at room temperature for 1 h; protein bands were visualized by enhanced chemiluminescence (Millipore, MA, USA) and analyzed by densitometry.

For immunoprecipitation, equal amounts (according to protein content) of cell lysates prepared as above were incubated with Trx1 antibody immobilized on protein A magnetic beads (Santa Cruz, CA, USA) at 4°C overnight. The pellet was washed five times with ice-cold NP40 (Beyotime, Shanghai, China); proteins were extracted with SDS-loading buffer and analyzed by immunoblotting with Trx1 and ASK1 antibodies.

### TrxR activity analysis in cells

TrxR activity in cervical cancer cells was determined by SC-TrxR assay as described by Cunniff *et al* [67]. Cells were incubated with different concentrations of B5 for 48 h. Cells were harvested and cell lysates were prepared as described above in the presence of protease inhibitors. Protein concentrations of the supernatants were determined by Bradford reagent. 25 μg of extract was incubated with 1 mM NADPH and 2 mM SC in TE buffer (50 mM Tris-HCl and 2 mM EDTA, pH 7.5) in a total volume of 100 μl. Samples of protein only, protein and NADPH, and protein and SC were used as controls, respectively. The reaction mixture was placed into 96-well microplates and monitored in 30-s intervals over a 20-min time period at 340 nm using a microplate reader (BioRad, USA). TrxR activity was expressed as mol NADPH consumed/min.

### Thioredoxin redox status assay

After the treatment with 4, 16, and 32 μM B5 for 48 h, 1 × 10^6^ CaSki and SiHa cells were lysed in 50 mM Tris/HCl, pH 8.3, 3 mM EDTA, 6 M guanidinium chloride, 0.5% Triton X-100 containing 50 mM iodoacetic acid [68]. After incubation at 37°C in the dark for 30 minutes, excess iodoacetic acid was removed using desalting column (Micro Bio-Spin, BIO RAD, USA). Oxidized and reduced Trx-1 were separated by non-reduced SDS-PAGE and immunoblotting.

### Quantitative real-time RT-PCR

CaSki and SiHa cells (2 × 10^5^ cells/well) were seeded into 6-well plates. After the treatment with 4, 16, and 32 μM B5 for 48 h, cells were harvested, and total RNA was isolated using PureLink™ Viral RNA/DNA Kits (Invitrogen). The quantitative PCR of Trx-1 and TrxR1 mRNA was determined using SYBR Green Realtime PCR Master Mix (Toyobo) as described by the manufacturer. Gene-specific primer pairs used in this study were as follows: TrxR sense 5′-TGTTGAATGAACAACTGTGC-3′ and TrxR antisense 5′-TCCTCAGCCAGTACATTGAC-3′, Trx sense 5′-CATA ACCAGCCATTGGCTATT-3′ and Trx antisense 5′-GCAT AATGTTTATTGTCACG-3′, GAPAH sense 5′-CACCCA GAAGACTGTGGATGG-3′ and GAPDH antisense 5′-GTCTACATGGCAACTGTGAGG-3′.

### Statistical analysis

All data are reported as mean ± SD of three independent experiments performed in triplicate. The differences between two treatment groups were analyzed by two-tailed unpaired Student's *t* test; three or more groups were compared by one-way ANOVA multiple comparisons. *P*-values < 0.05 were regarded as statistically significant.
